# A retrospective review of nine cases of atypical neurosyphilis

**DOI:** 10.3389/fmed.2025.1603596

**Published:** 2025-06-24

**Authors:** Ran Miao, Canglin Song, Wenjing Zhang, Xiaoyang Ma, Yi Zhang, Yuming Huang

**Affiliations:** Department of Neurology, Beijing Ditan Hospital, Capital Medical University, Beijing, China

**Keywords:** atypical neurosyphilis, mimicking, autoimmune encephalitis, brain magnetic resonance imaging, mesiotemporal lobe

## Abstract

**Background:**

Neurosyphilis may manifest in a form resembling autoimmune encephalitis, primarily characterized by cognitive impairment, psychiatric disorders, and seizures. Brain magnetic resonance imaging typically reveals unilateral or bilateral lesions in the mesiotemporal lobe and may also identify abnormalities in the inferior frontal lobe, parietal lobe, occipital lobe, and thalamus. Nevertheless, there are very few reports addressing this particular aspect.

**Methods:**

We conducted a retrospective review of the medical records of inpatients diagnosed with neurosyphilis who presented with cognitive impairment, psychiatric disorders, and seizures. All patients underwent lumbar puncture and cerebrospinal fluid (CSF) examination. A total of nine inpatients were consecutively enrolled from Beijing Ditan Hospital between July 2019 and December 2024.

**Results:**

Seven patients were male, while two patients were female. All patients presented with acute or subacute onset and had no fever prior to the onset. Four patients presented with seizures, two exhibited psychiatric and behavioral abnormalities, and three experienced memory decline. Throughout the course of the disease, a total of seven patients presented seizures. In the physical examination, all patients exhibited cognitive impairment. Regarding the CSF analysis, the initial results for one patient were inconclusive. The remaining patients demonstrated lymphocyte-predominant pleocytosis along with elevated CSF protein concentrations. One patient tested positive for the anti-CV2/CRMP5 antibody in both serum and CSF. Magnetic resonance imaging of the brain revealed that eight patients had sustained damage to the mesiotemporal lobe, while one patient demonstrated damage exclusively to the bilateral frontal and occipital lobes. Five patients exhibited sharp waves or sharp-slow waves on their electroencephalograms. All patients showed improvement in symptoms after antisyphilitic treatment for neurosyphilis.

**Conclusion:**

Neurosyphilis may manifest with symptoms associated with the limbic system, and brain magnetic resonance imaging often reveals unilateral or bilateral lesions in the mesiotemporal lobe that resemble those seen in autoimmune encephalitis. Early intervention with antisyphilitic treatment has proven to be effective. Clinicians should remain vigilant for such atypical presentations of neurosyphilis.

## Introduction

Neurosyphilis (NS) is a condition caused by the invasion of *Treponema pallidum* into the central nervous system (CNS), which can manifest at any time following infection. NS encompasses several forms, including asymptomatic neurosyphilis, symptomatic syphilitic meningitis, meningovascular neurosyphilis, intracranial gummas, general paresis, and tabes dorsalis (1). Some studies conducted during the antibiotic era have reported cases of atypical neurosyphilis; most patients in these reports exhibit cognitive impairments, psychiatric symptoms, and seizures that present similarly to herpes simplex virus encephalitis or autoimmune encephalitis (AE) (2). It is challenging to classify these cases within the previously mentioned typical clinical forms of neurosyphilis, including symptomatic syphilitic meningitis, meningovascular neurosyphilis, general paresis, and tabes dorsalis. Consequently, some scholars refer to this condition as atypical neurosyphilis. Brain magnetic resonance imaging (MRI) typically reveals unilateral or bilateral lesions in the medial temporal lobe characterized by high signal intensity on T2-weighted and fluid-attenuated inversion recovery (T2-FLAIR) sequences (3). In addition to abnormalities in the medial temporal lobe, MRI may also reveal signal changes in other regions such as the inferior frontal lobe, parietal lobe, occipital lobe, and thalamic area (4, 5). These lesions often show regression following treatment for NS. Early intervention for NS has proven to be highly effective. However, there exists a paucity of literature on this subject; most available reports are limited to case studies. Therefore, we undertook this retrospective study to analyze the clinical characteristics and laboratory findings associated with atypical NS.

## Methods

### Study design

We conducted a retrospective analysis of inpatient clinical records from the Department of Neurology at Beijing Ditan Hospital, Capital Medical University. Our review focused on inpatients diagnosed with NS who exhibited cognitive impairment, psychiatric disorders, and seizures between July 2019 and December 2024. To exclude other conditions mimicking NS, all patients underwent lumbar puncture. We collected comprehensive clinical and laboratory data, including age, sex, clinical manifestations, laboratory findings, imaging results, and electroencephalogram (EEG) readings. We used indirect immunofluorescence assay to detect for AE-related antibodies such as anti-Hu antibody, anti-Ri antibody, anti-Yo antibody, anti-NMDA receptor antibody, anti-AMPA1 receptor antibody, anti-AMPA2 receptor antibody, anti- GABA_B_ receptor antibody, anti-CASPR2 antibody, anti-LGI1 antibody, anti-GAD receptor antibody, anti-CV2/CRMP5 antibody, anti-IgLON5 antibody, anti-DPPX antibody, anti-GlyR1 antibody, anti-DRD2 antibody, anti-mGluR5 antibody, and anti-mGluR1 antibody. The detection of pathogens in blood and cerebrospinal fluid (CSF) included testing for human immunodeficiency virus, herpes simplex virus, cytomegalovirus, toxoplasma, varicella-zoster virus and rubella virus. The most commonly used nontreponemal test to diagnose NS from CSF is the Venereal Disease Research Laboratory (VDRL) test. However, it has low sensitivity and its procedure is complicated and time-consuming. Furthermore, the Chinese Food and Drug Administration has not approved any commercial VDRL reagents for use in CSF analysis. Recent studies suggest that toluidine red unheated serum test (TRUST) could serve as an alternative diagnostic tool for NS when VDRL is unavailable (6, 7). Following a detailed evaluation based on established diagnostic criteria, a total of nine subjects were included in this study. This research was approved by the ethics committee of Beijing Ditan Hospital.

### Diagnostic criteria

The diagnostic criteria for NS were established based on the guidelines provided by the Centers for Disease Control and Prevention in both the United States and Europe (8, 9). A diagnosis of syphilis was confirmed when both serum *Treponema pallidum* particle agglutination (TPPA) or fluorescent treponemal antibody absorption (FTA-ABS) tests, as well as the TRUST test, yielded reactive results. Syphilis at any stage with reactive TRUST in the CSF was confirmed as NS. In cases where CSF-TRUST results were negative, a diagnosis of NS required the presence of both of the following abnormal findings: (a) a positive result for either CSF TPPA or FTA-ABS; and (b) an elevated white blood cell count (WBC) in CSF (CSF WBC > 5 cells/μL) or increased protein levels (> 45 mg/dL), without any other known causes accounting for these abnormalities. Patients were excluded from this study if their clinical manifestations could not be attributed to NS or if they had concurrent CNS infections.

## Results

Five patients were excluded from our study due to the inability to obtain the initial CSF TRUST test results. The five patients exhibited symptoms associated with the limbic system. Brain MRI revealed involvement of the medial temporal lobe, and lumbar puncture results indicated elevated protein or cell counts in the CSF. However, the five patients in question did not initially seek treatment at our hospital. Due to the lack of CSF TRUST test results, a re-evaluation of the lumbar puncture findings revealed that the CSF TRUST test was negative, thereby precluding a definitive diagnosis of neurosyphilis.

A total of nine patients were enrolled in the study, all of whom tested positive for either TPPA or FTA-ABS in both serum and CSF. Among these patients, seven were male and two were female. All individuals presented with an acute or subacute onset of symptoms and reported no fever prior to the onset. The ages at which symptoms began ranged from thirty-two to sixty-four years. The duration from the onset of initial symptoms to penicillin treatment for patient 1 through 9 was as follows: 4 months, 21 months, 11 days, 12 months, 3 months, 6 months, 14 days, 6 months, and finally, 12 days. Four patients exhibited seizures, while two presented with psychiatric and behavioral abnormalities, and three experienced memory decline. Throughout the course of the disease, a total of seven patients had seizures; six experienced generalized tonic–clonic seizures, whereas one patient had focal seizures (Table 1).

**Table 1:** Demographic, clinical information and laboratory data of all patients.

No	Sex Age Form of onset	Clinical manifestations Physical examination	Serum TRUST titer CSF TRUST titer AE-related antibody	CSF WBC count, /ul Monocyte proportion (%) CSF protein concentration, mg/dl
1	M 32 Subacute	Psychiatric, seizures Memory and calculation abilities decline	1:32 1:8 Negative	62 93.5 95
2	M 30 Acute	Seizures Memory and orientation decline	1:16 1:1 Anti-CV2/CRMP5 antibody in both serum and CSF	NA NA NA
3	M 55 Acute	Seizures Memory, calculation ability, orientation, and comprehension judgment decline	1:64 1:2 Negative	84 96 118.8
4	M 56 Subacute	Psychiatric, seizures Memory decline	1:128 1:1 Negative	32 NA 81
5	M 51 Acute	Cognitive impairment Slow reaction, memory decline	1:32 1:2 Negative	120 97 140.8
6	F 52 Acute	Cognitive impairment, seizures Memory decline	1:64 1:4 Negative	87 NA 74
7	M 35 Acute	Seizures Memory decline, the tendon reflexes in both lower limbs were not elicited	1:128 1:8 Negative	92 95 87
8	F 64 Subacute	Cognitive impairment, psychiatric Memory decline, Argyll Robertson pupil	1:4 Negative Negative	18 89 71.9
9	M 61 Acute	Seizures Slow reaction, memory decline	1:64 1:2 Negative	40 90 47.3

TRUST, toluidine red unheated serum test; WBC, white blood cell count; CSF, cerebrospinal fluid; AE, autoimmune encephalitis; NA, not available.

In the physical examination, all patients demonstrated cognitive impairment. Patient 8 exhibited an Argyll Robertson pupil, whereas patient 7 did not display tendon reflexes in either lower limb (Table 1).

In laboratory examinations, serum TRUST tests were positive for all patients, with titers ranging from 1:4 to 1:128. Regarding CSF analysis, the initial lumbar puncture results for one patient’s CSF cell and protein concentration were inconclusive. The remaining patients demonstrated CSF pleocytosis along with elevated protein concentrations; three of these patients did not show a proportion of mononuclear cells, whereas six others exhibited lymphocyte-predominant pleocytosis. Notably, only one patient’s CSF TRUST result was negative, while the TRUST titers in the CSF of other patients ranged from 1:1 to 1:8 (Table 1).

In the initial AE-related antibody testing, patient 2 displayed a strong positive result for anti-CV2/CRMP5 antibodies in both serum and CSF samples.

**Figure 1:**
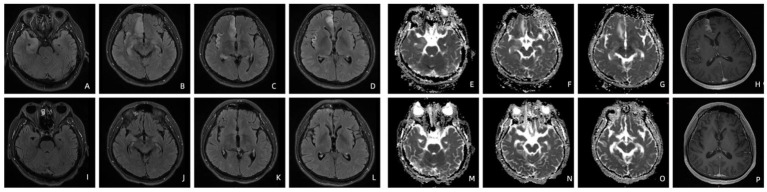
T2-FLAIR imaging of patient 1 before **(A–D)** and four months after treatment **(I–L)**. The ADC **(E–G)** and contrast-enhanced T1-weighted imaging **(H)** of patient 1 before and four months after treatment **(M–P)**.

Patient 1’s T2-FLAIR imaging revealed hyperintensities in the right superior frontal gyrus, rectus gyrus, insula, and bilateral hippocampi. The apparent diffusion coefficient (ADC) imaging demonstrated hyperintensity in the right rectus gyrus and hippocampi. Contrast-enhanced T1-weighted imaging indicated enhancement in the right insula and superior frontal gyrus. Following penicillin treatment, the hyperintensities observed on T2-FLAIR in the right rectus gyrus, insula, and bilateral hippocampi completely resolved, with a significant reduction noted in the hyperintensity of the right superior frontal gyrus (Figure 1). Both ADC and contrast-enhanced T1-weighted images returned to normal appearances (Figure 1).

Patient 2’s initial T2-FLAIR brain MRI upon admission revealed high-intensity lesions in the right mesiotemporal lobe and parietal lobe, along with signs of cerebral atrophy. The ADC imaging showed hyperintensities localized to both regions as well. Contrast-enhanced T1-weighted imaging revealed patchy enhancement within the right parietal lobe. After undergoing penicillin treatment, there was a marked decrease in abnormal signals within the right parietal lobe; however, abnormal signals persisted in the right temporal lobe. The ADC imaging continued to show high intensity associated with the right mesiotemporal lobe while contrast-enhanced T1-weighted image appeared normal.

Both Patient 3 and Patient 5 exhibited high-intensity lesions located in the left mesiotemporal lobe on their respective T2-FLAIR brain MRIs. The ADC imaging confirmed hyperintensities present within this region; however, contrast-enhanced T1-weighted imaging appeared normal. Post-penicillin treatment evaluations demonstrated complete resolution of both T2-FLAIR and ADC hyperintensities on subsequent brain MRIs.

Patient 4’s T2-FLAIR brain MRI revealed high-intensity lesions located in the right mesiotemporal lobe. The ADC imaging exhibited hyperintensity in the right mesiotemporal region. The contrast-enhanced T1-weighted image demonstrated a normal appearance. Following penicillin treatment, subsequent brain MRI indicated only mild persistence of the abnormal signal within the right mesiotemporal lobe on T2-FLAIR imaging, while the ADC image returned to normal.

**Figure 2:**
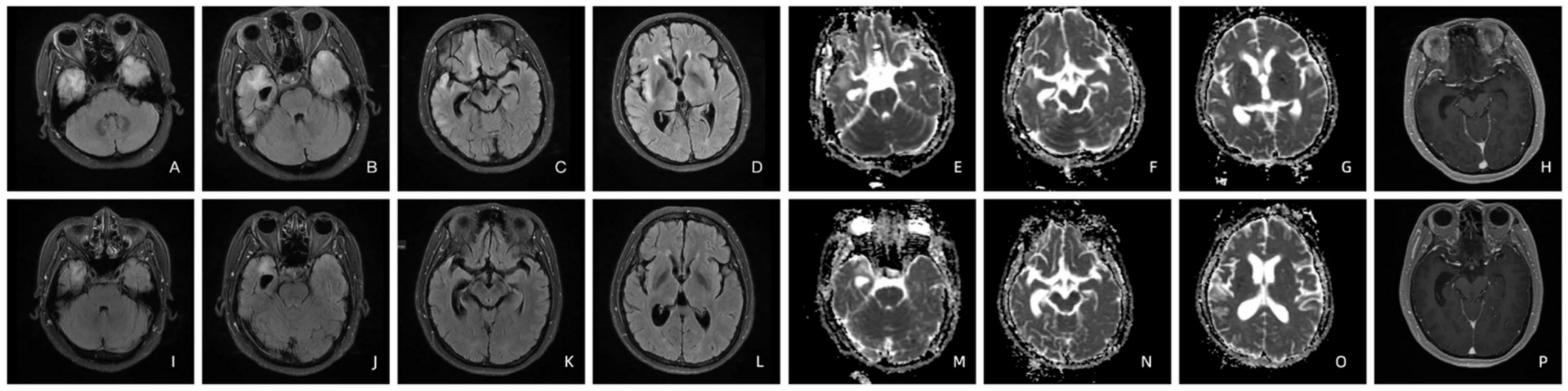
T2-FLAIR imaging of patient 6 before **(A–D)** and four months after treatment **(I–L)**. The ADC **(E–G)** and contrast-enhanced T1-weighted imaging **(H)** of patient 6 before and four months after treatment **(M–P)**.

Patient 6’s T2-FLAIR brain MRI showed high-intensity lesions affecting the right rectus gyrus, insula, and bilateral mesiotemporal lobes. Hyperintensity was evident in right rectus gyrus, mesiotemporal lobe and insula on the ADC imaging as well. The contrast-enhanced T1-weighted imaging displayed enhancement specifically in the right mesiotemporal lobe. After administration of penicillin, brain MRI results illustrated a significant reduction in hyperintensity on T2-FLAIR imaging, with only mild persistence of abnormal signals noted bilaterally in the mesiotemporal lobes (Figure 2). Notably, the hyperintensities previously observed in the right rectus gyrus and insula have completely resolved on the ADC imaging. Furthermore, there was a significant reduction in the range of hyperintensity within the right hippocampus as seen on the ADC imaging. The contrast-enhanced T1-weighted imaging once again demonstrated a normal appearance (Figure 2).

Patient 7’s T2 FLAIR brain MRI identified high-intensity lesions present within both the right mesiotemporal lobe and occipital lobe. Hyperintensity was also observed in these areas on an ADC imaging. Furthermore, patchy enhancement appeared within the right occipital lobe on contrast-enhanced T1-weighted imaging. Subsequent to penicillin treatment, all signs of hyperintensities were found to be completely resolved across all modalities: T2-FLAIR, ADC imaging, and contrast-enhanced T1-weighted imaging.

Patient 8’s T2 FLAIR brain MRI revealed high-intensity lesions in the bilateral mesiotemporal lobes. The ADC imaging exhibited hyperintensity in the right mesiotemporal lobe, while the contrast-enhanced T1-weighted imaging appeared normal. Following penicillin treatment, subsequent brain MRI demonstrated a significant reduction in T2-FLAIR hyperintensity, with only mild persistence of abnormal signals located in the right mesiotemporal lobe. The ADC imaging indicated that the hyperintensity within the right mesiotemporal lobe had decreased.

Patient 9’s T2 FLAIR brain MRI displayed high-intensity lesions in both frontal lobes’ cortices and the right occipital lobe cortex. Both ADC imaging and contrast-enhanced T1-weighted imaging showed normal appearances. After penicillin treatment, follow-up brain MRI revealed complete resolution of the T2-FLAIR hyperintensities.

The electroencephalogram exhibited nonspecific findings, which differ from previous literature as no periodic lateralized epileptiform discharges-like manifestations were observed. With the exception of patient 7, electroencephalography revealed localized slow or spiked waves in all other patients (Table 2).

**Table 2:** Findings from the follow-up of electroencephalogram.

No	EEG
1	Initial EEG: intermittent sharp waves and sharp-slow waves in the right frontopolar, anterior temporal, middle temporal, and posterior temporal regions Four months later: a limited presence of paroxysmal irregular slow waves Ten months later: normal Fourteen months later: normal Eighteen months later: normal Twenty-two months later: a limited presence of paroxysmal irregular slow waves
2	Initial EEG: intermittent right frontopolar and temporal slow waves Five months later: sharp-slow waves are prominently observed in the right occipital and posterior temporal regions, accompanied by multi-spike-slow waves discharges Ten months later: occasional paroxysmal episodes of slow waves are observed in the anterior, middle, and posterior temporal regions on the left side Twenty-eight months later: sharp-slow wave discharges are observed in the right occipital, anterior, middle, and posterior temporal regions. These discharges may also involve the right frontal pole and adjacent frontal areas Thirty-six months later: Sharp waves and sharp-slow waves were observed in the right frontal pole, as well as in the frontal, anterior temporal, and middle temporal regions
3	Initial EEG: paroxysmal slow waves activity across all leads Four months later: normal Thirty months later: normal
4	Initial EEG: sharp waves and sharp slow waves in the right frontopolar, frontal, occipital, anterior, and middle temporal regions Four months later: numerous sharp waves, along with sharp-slow waves, are prominently observed in the right occipital and posterior temporal regions Eight months later: in the right occipital and posterior temporal regions, there is a prominent presence of both sharp waves and sharp-slow waves Twenty-four months later: in the right occipital and posterior temporal regions, a significant number of sharp waves and sharp-slow wave patterns can be observed Twenty-eight months later: sharp waves, sharp-slow waves, and polyspike-slow waves discharges were observed in the right frontal pole, as well as in the frontal, central, anterior, middle, and posterior temporal regions. Additionally, these discharges were noted in both the frontal midline and central midline areas
5	Initial EEG: paroxysmal slow waves activity across all leads Four months later: normal
6	Initial EEG: sharp slow waves in the right central, parietal, middle, and posterior temporal regions Four months later: atypical sharp waves and sharp-slow waves were observed in the right anterior and middle temporal regions
7	Initial EEG: normal Four months later: normal Eight months later: normal
8	Initial EEG: paroxysmal slow waves activity across all leads Five months later: normal
9	Initial EEG: sharp waves and sharp-slow waves were observed in the right frontal pole, frontal lobe, anterior, middle and posterior temporal regions Four months later: paroxysmal irregular slow waves Fight months later: normal

We utilized the Mini-Mental State Examination (MMSE) and Montreal Cognitive Assessment (MoCA) scales to evaluate cognitive function. Only one patient did not complete the baseline assessment (patient 3), while the results for the other patients indicated a decline in cognitive function. The baseline scores of the MMSE and the MoCA for the remaining eight patients were as follows: 26/18 (patient 1), 23/19 (patient 2), 22/18 (patient 4), 21/19 (patient 5), 21/18 (patient 6), 18/8 (patient 7), 20/20 (patient 8), and 21/21 (patient 9).

For treatment, we administered intravenous penicillin at a dosage of four million units every four hours, followed by intramuscular penicillin G benzathine for three weeks. Due to the positive presence of anti-CV2/CRMP5 antibody in patient 2, we administered intravenous immunoglobulin therapy concurrently. The other eight patients did not receive treatment with intravenous steroids therapy, intravenous immunoglobulin, or plasmapheresis therapy.

We conducted follow-up lumbar punctures on these nine patients. Patients 1, 2, 3, 4, 6, 8, and 9 demonstrated that CSF-related indicators returned to normal levels at the following time points post-treatment: Patient 1 at month twenty-eight; Patient 2 at month ten; Patient 3 at month thirty-six; Patient 4 at month four; Patient 6 at month twenty-four; Patient 8 at month twelve; and Patient 9 at month eighteen. However, patients 5 and 7 were lost to follow-up after six months and eight months of monitoring, respectively. Subsequent follow-up assessments enabled the discontinuation of intravenous penicillin treatment once CSF-related indicators normalized.

Among the seven patients who presented seizures, four individuals (patients 3, 6, 7, and 9) successfully reduced and ultimately discontinued their oral anti-seizure medications without experiencing any clinical seizures. In contrast, three patients (patients 1, 2, and 4) necessitated long-term maintenance therapy with oral anti-seizure medications. In Table 2, we provide a detailed account of the electroencephalographic follow-up results for nine patients.

Patient 1 was initially administered oral sodium valproate and oxcarbazepine to manage the epileptic seizures. Within four months following the onset of the disease, the patient continued to experience unprovoked seizures; consequently, levetiracetam was introduced as an additional therapy. From four months to twenty-two months following the onset of symptoms, the patient remained clinically seizure-free, and EEG results were within normal limits. At the twenty-two-month follow-up, the levels of CSF WBC and protein detected through lumbar puncture in the patient returned to within the normal range. However, a recurrence of unprovoked seizures was observed twenty-eight months after the onset of the disease. The laboratory examination revealed normal CSF protein and cell counts, negative CSF TRUST results, weakly positive serum anti-CV2/CRMP5 antibody, and positive CSF anti-CV2/CRMP5 antibody. Brain MRI showed high-intensity lesions in the right prefrontal lobe and cingulate cortex. We initiated treatment with intravenous immunoglobulin along with oral valproate, oxcarbazepine, and levetiracetam. Following this intervention, his seizure symptoms improved significantly; brain MRI demonstrated complete resolution of T2-FLAIR hyperintensities. However, the patient has continued to experience seizures triggered by fever or emotional agitation, occurring at a frequency of twice per year.

Patient 2 was initially treated with a combination of oxcarbazepine and lacosamide, during which no clinical epileptic seizures were observed; consequently, lacosamide was gradually tapered and eventually discontinued. At the ten-month follow-up, the levels of CSF WBC and protein returned to within normal limits. Furthermore, the CSF TRUST test yielded a negative result. However, during the third year of follow-up, the patient did not exhibit any clinical seizures; however, interictal epileptiform discharges remained evident on the EEG. Consequently, we gradually reduced the dosage of oxcarbazepine.

Patient 4 received levetiracetam for the treatment of epilepsy. At the four-month follow-up, the levels of CSF WBC and protein identified through lumbar puncture in the patient returned to within normal limits. Furthermore, the CSF TRUST test produced a negative result. During the initial eight months, the patient was treated with oral levetiracetam alone and did not experience any clinical seizures. However, after eight months from onset, the patient began to occasionally exhibit focal seizures during episodes of fever. Consequently, oxcarbazepine was introduced as an additional oral treatment; nevertheless, focal seizures continued to occur in conjunction with fever. Notably, interictal epileptiform discharges remained evident on the EEG.

We propose the hypothesis that patients 2 and 4 may have developed postencephalitic epilepsy.

Upon the conclusion of penicillin treatment, all patients, with the exception of patient 2, exhibited significant improvements in their scores on both the MMSE and MoCA scales (Table 3).

**Table 3:** Follow-up of cognitive function.

No	Baseline MMSE/MOCA	Four months later MMSE/MOCA	Eight months later MMSE/MOCA
1	26/18	27/25	28/27
2	23/19	23/12	22/18
3	NA	26/19	28/26
4	22/18	26/18	27/21
5	21/19	25/24	NA
6	21/18	28/23	29/27
7	18/8	22/15	22/17
8	20/20	25/20	25/20
9	21/21	25/23	28/23

MMSE, Mini-Mental State Examination; MOCA, Montreal Cognitive Assessment; NA, not available.

## Discussion

AE refers to a form of encephalitis that is mediated by autoimmune mechanisms. This includes encephalitis syndromes associated with anti-neuronal surface or synaptic protein antibodies, as well as those linked to anti-intracellular neuronal protein antibodies. According to the diagnostic criteria for possible AE proposed in 2016: ① subacute onset (rapid progression of less than 3 months) of working memory deficits (short-term memory loss), altered mental status, or psychiatric symptoms. ② At least one of the following: new focal CNS findings, seizures not explained by a previously known seizure disorder. CSF pleocytosis (white blood cell count of more than five cells per mm3), MRI features suggestive of encephalitis (brain MRI hyperintense signal on T2-FLAIR sequences highly restricted to one or both medial temporal lobes, or in multifocal areas involving grey matter, white matter, or both compatible with demyelination or inflammation). ③ Reasonable exclusion of alternative causes (10).

The patients we reported all meet the diagnostic criteria for possible AE. However, the patient was subsequently diagnosed with NS following positive serological and CSF specific antigen test results for syphilis. NS is one of the most important differential diagnoses for AE. Due to the widespread use of antibiotics, cases of NS with atypical clinical presentations reports have been increasing; among these cases, NE similar to AE is rare and primarily documented as individual instances.

Patient 2 meets the diagnostic criteria for both NS and anti-CV2/CRMP5 encephalitis, which we consider to be overlapping conditions. We administered penicillin and intravenous immunoglobulin concurrently while also utilizing oral antiepileptic medications. Although the patient’s epilepsy was effectively controlled, cognitive recovery was suboptimal, and anti-CV2/CRMP5 antibodies remained detectable even after clinical improvement, a finding consistent with previous literature (11).

Patient 1 experienced a seizure recurrence two years later. At that time, lumbar puncture results did not fulfill the diagnostic criteria for NS; however, they did meet those for anti-CV2/CRMP5 encephalitis. Immunoglobulin therapy proved effective; following treatment, seizure symptoms were controlled and brain MRI lesions showed significant reduction.

CV2/CRMP5 antibody-associated paraneoplastic neurological syndrome (PNS) is a relatively common variant of PNS characterized by its distant effects from tumors rather than direct tissue or organ invasion. This condition has an immune-mediated etiology, with patients frequently exhibiting specific neural antibodies. In the 2021 update of PNS diagnostic criteria, it was classified as a high-risk antibody associated with small-cell lung cancer and thymoma (12). Clinical manifestations are diverse and may include limbic encephalitis, ocular symptoms, chorea, cerebellar ataxia, myelopathy, and peripheral neuropathy (13). We conducted relevant examinations and follow-ups on patients 1 and 2 but found no clear indications of tumors. Currently, there appears to be no existing literature addressing the relationship between NS and anti-CV2/CRMP5 encephalitis; thus, further research may be warranted in this area in the future.

Radiologically, it is essential to differentiate NS characterized by mesiotemporal lobe lesions from viral encephalitis and AE. The “knife-cut sign” serves as a more distinctive feature associated with herpes simplex encephalitis, while the manifestations of NS are comparatively rare. On MRI, viral encephalitis typically presents with edema at the lesion site accompanied by significant mass effect. Additionally, it may demonstrate mild brain enhancement and subacute intracerebral hemorrhage. In contrast, NS generally exhibits atrophy at the lesion site, with bleeding being uncommon and no evident mass effect.

Regarding AE, MRI findings in cases of anti-Hu, anti-Ma/Ta, anti-GAD, anti-GABAB receptor, anti-AMPA receptor, and anti-VGKC encephalitis typically reveal T2-FLAIR hyperintensity in the mesial temporal lobes; however, other presentations are less likely (14).

The pathological mechanisms and changes associated with NS lesions in the medial temporal lobe remain inadequately understood. Given their reversibility and the high apparent diffusion coefficient signals observed in certain cases, existing literature suggests that these signal alterations may arise from a combination of edema and gliosis, or may be linked to increased permeability of the blood–brain barrier alongside inflammatory responses within small vessel meninges (15). This interplay can result in both vasogenic and cytotoxic edema.

In the early stages of the lesion, syphilitic microvascular changes contribute to altered vascular permeability; thus, it is likely that initial manifestations are primarily due to vasogenic edema (16). As the lesion progresses into later stages, edema combined with vasculitis induces ischemia and hypoxia within brain tissue, ultimately leading to cytotoxic edema. During this initial phase, treatment remains reversible and compensatory, often resulting in alleviation of temporal lobe lesions.

However, once the condition progresses to a chronic stage characterized by neuroglial hyperplasia and microinfarctions—accompanied by atrophy of the temporal lobe on the affected side as well as enlargement of the temporal horn of the lateral ventricle—the prognosis for treatment outcomes significantly deteriorates. In advanced stages of syphilis, lesions become irreversible; pronounced atrophy of the temporal lobe (including hippocampal atrophy) serves as an indicator of poor prognosis (17).

Patients diagnosed with syphilitic encephalitis typically respond favorably to early standardized penicillin treatment, often resulting in the complete resolution of certain clinical symptoms. Following treatment, it is essential to re-evaluate CSF every six months. Any abnormalities detected in the CSF may resolve within two years; however, if they persist, a renewed course of standardized treatment will be necessary.

## Conclusion

Neurosyphilis may present in a manner that resembles autoimmune encephalitis, primarily characterized by cognitive impairment, psychiatric disorders, and seizures. Magnetic resonance imaging of the brain frequently reveals unilateral or bilateral lesions in the mesiotemporal lobe. Early intervention with antisyphilitic treatment has demonstrated efficacy. Clinicians should maintain a high level of vigilance for such atypical manifestations of neurosyphilis.

## Data Availability

The raw data supporting the conclusions of this article will be made available by the authors, without undue reservation.
